# Complications following endoscopic transsphenoidal surgery for pituitary adenoma—special focus on intrasellar pressure

**DOI:** 10.1007/s00701-025-06495-7

**Published:** 2025-03-19

**Authors:** Gabriel Simander, Per Olof Eriksson, Sara Viirola, Peter Lindvall, Lars-Owe D. Koskinen

**Affiliations:** 1 Department of Clinical Sciences-Neurosciences, Umeå University, Umeå, Sweden; 2 Department of Surgical Sciences, Otorhinolaryngology and Head and Neck Surgery, Uppsala University, Uppsala, Sweden

**Keywords:** Intrasellar pressure, Pituitary adenoma, Endoscopic transsphenoidal, Complications

## Abstract

**Purpose:**

The aim of this study was to explore risk factors for intraoperative events and postoperative complications of endoscopic transsphenoidal surgery (ETS) for pituitary tumors, and the role of intrasellar pressure (ISP) in relation to complications.

**Methods:**

The study was a single-center, retrospective, consecutive, observational study, with ISP data collected prospectively. After exclusions, the study population encompassed 69 patients. All had ISP measured intraoperatively during ETS for a pituitary adenoma and underwent standardized postoperative observations and follow-up. Data on complications within 3 months after surgery and some risk factors were collected retrospectively.

**Results:**

Decreased risk of postoperative cerebrospinal fluid leakage was seen with higher age. Large tumor volume was associated with higher risk of intraoperative events. ISP was not associated with complication frequency, but patients with ISP > 20 mmHg had increased frequency of postoperative epistaxis.

**Conclusion:**

This study confirms earlier findings of low age as a possible risk factor for postoperative cerebrospinal fluid leakage. Tumor volume is suggested to be associated with higher complication risk. ISP does not seem to be a significant risk factor for intraoperative events or postoperative complications following ETS. Predictive risk factors for surgical complications after ETS are still not satisfactorily explained and heterogeneous definitions of complications are problematic in this context.

## Introduction

Pituitary adenoma account for 15% of intracranial tumors and most are benign [20]. Symptoms are caused by hormonal hypersecretion, hypopituitarism, and mass effects of the tumor, resulting in visual field defects and headaches. However, pituitary adenomas are not seldom asymptomatic and may go unrecognized for many years [23].

The majority of symptomatic tumors are treated with transsphenoidal surgical resection, with the exception being prolactinomas, for which pharmacological treatment is the first choice [23]. The development of endoscopic transsphenoidal surgery (ETS) has improved visibility in the surgical area and promoted quicker recovery for patients [4, 6].

Some of the most common postoperative complications that can follow ETS are anterior pituitary insufficiency, diabetes insipidus, cerebrospinal fluid (CSF) leakage, epistaxis, meningitis, and intrasellar hematoma [19, 20]. Multiple studies have attempted to find risk factors associated with postoperative complications, and some correlations have been found between older age, macroadenomas, and tumor invasiveness [5, 10]. However, due to the methodological quality of the current literature, risk factors for complications cannot be comprehensively quantified [18].

Intracranial pressure (ICP) is widely used as a diagnostic tool today, and it is well known that increased ICP can negatively impact cerebral circulation after traumatic brain injury, leading to increased morbidity [21]. Intraoperatively measured intrasellar pressure (ISP) has been presented in only a few previous studies. Some have found associations between ISP and tumor anatomy and hormonal disturbance, indicating that ISP measurement could potentially have clinical relevance [1, 9, 16, 17, 24, 25]. One could hypothesize that ISP is associated with intraoperative events and postoperative complications. Thus far, no study has explored the association between complication risk and ISP. As of today, there is no concise way to predict possible complications in conjunction with pituitary surgery, and knowledge of predictive factors is needed to improve risk analysis and patient counseling in this regard [18].

This study aimed to describe overall intraoperative events and postoperative complications in ETS in relation to anatomical and pathophysiological mechanisms and bridge the knowledge gap regarding ISP as a risk factor for complications. We hypothesized that elevated ISP could have negative effects on the pituitary gland and surrounding structures, which could lead to an increased frequency of intraoperative events and postoperative complications.

## Methods and materials

### Study population

The study was a single-center, consecutive, retrospective, observational study with ISP data collected prospectively. Patients who had underwent surgery for a pituitary tumor, except for Cushing’s disease, at Umeå University Hospital, Sweden between 2009–2015, were included in the study. All of them had pre-resection ISP measured intraoperatively. The reason behind not including patients with Cushing’s disease is that since these procedures are often more challengeable, not seldomly because of a small sella containing a microadenoma, the ISP measurement practice was not performed during these surgeries. Patients were qualified for surgery in accordance with standard clinical practice. This resulted in a consecutively collected population of 100 patients.

### Exclusions

Exclusion criteria were as follows:

- Previous pituitary surgery
- Non-endoscopic operation method
- Non-adenoma tumor diagnosis

Out of the 100 included patients, 6 were excluded due to having a diagnosis other than pituitary adenoma, 3 due to previous pituitary surgery, and 22 due to use of a non-endoscopic operation method. These exclusions were made as they were assessed to potentially affect complication rates, and to obtain as homogenous a study population as possible. We decided to include only patients who underwent ETS, as this is the operation technique used today, making evaluation thereof a priority. After exclusions, the study population consisted of 69 patients.

### Surgery and ISP measurement

ETS was the surgical approach. All procedures during these years (2009–2015) were performed with one neurosurgeon as the main surgeon responsible. Surgery was performed under standard neuro-anesthesia with oral intubation. Patients were normo-ventilated with pCO_2_ levels at 4.6–5.5 kPa. ISP measurements were included as part of the standardized surgical procedure at the department and measured before the start of tumor resection using an ICP monitoring device (Codman® MicroSensor™, Codman & Shurtleff Inc, Raynham, MA, USA). This device’s accuracy is well-documented, and it is used clinically as a standard method for ICP monitoring [13–15]. A minor bone opening, about 1.5 mm in diameter, was made with a high-speed drill. This was followed by a sharp cut of the capsule without allowing any tissue loss. After calibration, the sensor was inserted into the sellar room with particular attention paid to preventing leakage of intrasellar content. ISP values were determined after pressure fluctuations had settled, in the majority of cases approximately 30 s after insertion. After ISP measurement and removal of the Codman® MicroSensor, the operation proceeded with further removal of bone from the sellar floor and tumor resection. Thereafter, the sellar floor was routinely sealed using dural substitute, plate of synthetic bone substitute and fibrin sealant. The nasoseptal tissue was preserved for an eventual nasoseptal flap, but not used during primary surgery if not indicated by a heavy CSF leakage.

### Postoperative management

Patients were observed for at least 12 h in the neuro-postoperative care unit, and then at the neurosurgical ward until at least 6 days after surgery. Before discharge, all patients were examined by an ear-nose-throat physician and hormonally evaluated by an endocrinologist. Urine production was followed closely for at least the first 2 postoperative days. Clinical neurological examinations and measurements of body temperature were performed daily during the entire admission period. Laboratory tests, including of C-reactive protein, electrolytes, hemoglobin, and white blood cell count, were performed daily for the 2 days after surgery, and then repeated if needed. Continuous blood pressure monitoring was performed at the neuro-postoperative care unit, with the goal of keeping systolic blood pressure levels below 160 mmHg.

### Data collection

Data regarding intraoperative events and postoperative complications and risk factors were collected retrospectively from the included patients’ medical records from the Departments of Neurosurgery and Ear, Nose, and Throat Diseases. The reviewed medical records encompassed inpatient care in conjunction with the surgery, postoperative follow-ups, and, when applicable, additional consultations or hospital re-admissions in direct conjunction (≤ 3 months) with the operation.

Preoperative radiological images (preoperative magnetic resonance imaging with and without gadolinium contrast) were used to measure the tumor diameter axes (coronal, craniocaudal, anteroposterior), and calculate the tumor volume using Automatic Sectra Volume Tool (Sectra Workstation, IDS7, v. 23.1). The growth pattern and invasiveness of the tumors were analyzed and all tumors were classified using the Knosp and SIPAP classification [2, 8, 12].

### Definition of intraoperative events and postoperative complications

Intraoperative events were defined as bleeding, CSF leakage, and difficulty of extraction due to tumor consistency. We characterized postoperative complications as epistaxis, intrasellar or intracerebral hematoma, CSF leakage, postoperative infection, new neurological symptoms, and unusual heavy headache during inpatient care or soon thereafter. As medical records from inpatient care were reviewed, other possible complications that could be associated with the surgery were also recorded.

#### Intraoperative bleeding

Intraoperative bleeding was defined as bleeding from the cavernous sinus, general heavy bleeding, or significant bleeding from some other location that was mentioned in the operative report. Excessive blood loss was defined as more than 500 ml bleeding during the operation.

#### Intraoperative CSF leakage

We assumed that an intraoperative CSF leakage had occurred if it was mentioned in the operative report.

#### Difficulty of extraction due to tumor consistency

Abnormal tumor consistency could complicate tumor resection. Tumor consistency described as anything other than soft, non-compact, grainy, or normal – for instance firm or hard – was defined as atypical.

#### Postoperative epistaxis

Postoperative epistaxis was any case of epistaxis that required some ear-nose-throat intervention (e.g., tamponade or diathermy), at any point during inpatient treatment or closely thereafter. This included epistaxis that occurred during manipulation in the nasal cavity, such as tamponade removal, as well as spontaneous epistaxis.

#### Intracranial hematoma

Intracranial hematoma was confirmed with computed tomography and/or magnetic resonance tomography. Both intrasellar, intracerebral or other intracranial hematomas were included.

#### Postoperative CSF leakage

We defined CSF leakage as leakage that was diagnosed by a physician and required some kind of intervention, such as prescribed bed rest, lumbar drainage, or fistula repair operation.

#### Postoperative infection

Postoperative infections included those that occurred in the head-neck area and required antibiotic treatment. These patients were diagnosed by a senior infectious disease specialist with special interest in central nervous system infections.

#### New neurological symptoms

A patient was classified as having new neurological symptoms if they developed some new neurological deficit postoperatively. If a patient was minimally neurologically impaired at some point during postoperative inpatient care, but this was reversed to the patient’s normal status before discharge, it was not considered a new neurological symptom in the data.

#### Postoperative headache

We defined postoperative headache as any kind that was more severe than that expected after transsphenoidal resection of a pituitary tumor. We assumed that this was at hand if postoperative headache was mentioned in the discharge summary.

#### Clinically significant complications

We included all adverse events that occurred intra- and postoperatively. However, many of these are to be expected in ETS and have not always been defined as complications in previous studies. For that reason, we grouped clinically significant complications, to present complication rates comparable to those of other studies on postoperative complications. In this group, we included intracranial hematoma, CSF leakage requiring lumbar drainage or fistula repair operation, meningitis, and any permanent postoperative neurological deficit.

### Definitions of risk factors

#### Increased bleeding risk

We defined increased bleeding risk as treatment with anticoagulative medication and/or intake of nonsteroidal anti-inflammatory drugs within one day before surgery. One patient with need of preoperative platelet transfusion was also included here.

#### Increased infection risk

We defined increased infection risk as treatment with immunosuppressive medication, repeated infection problems prior to surgery, and/or having diabetes mellitus.

#### Acute apoplexy

The definition of acute apoplexy was the combination of rapid onset or worsening of symptoms together with radiological signs of pituitary apoplexy.

#### Parasellar invasive

Tumors were considered parasellar invasive if they were defined as Knosp parasellar grade 3–4.

Other risk factors included were age, sex, ISP, active smoking at time of surgery, and tumor volume.

### Statistics

Differences between two groups of subjects were studied using Student’s t-test for continuous variables and the chi-square test for nominal data. Results are presented as means ± standard deviations. Multivariate logistic regression models were applied to analyze the separate risk factors’ impact on complications. Results are presented as unit odds ratios (ORs). Data analysis was performed in the statistical software JMP version 15.1 (SAS Institute Inc., Cary, NC, USA). P-values < 0.05 were considered significant.

### Ethics

The study was approved by the Swedish Ethical Review Authority. It was conducted in accordance with the World Medical Association’s Declaration of Helsinki, Ethical Principles for Medical Research Involving Human Subjects.

## Results

### Characteristics

The study population after exclusions consisted of 69 patients. Basic tumor characteristics including size, anatomical classification and biochemical diagnoses are presented in Tables 1, 2, and 3. The distribution of the analyzed risk factors including age, sex, ISP, parasellar invasiveness, apoplexy, smoking, increased bleeding risk and increased infection risk are presented in Table 4.

**Table 1:** Tumor size

		Mean	95% CI	Min	Max
Tumor volume (cm^3^)		5.6	4.4–6.8	0.7	30.4
Coronal diamater (mm)		18.7	17.6–19.5	7.5	36
Anteroporterior diameter (mm)		16.7	15.4–18.0	7.5	39.0
Craniocaudal diameter (mm)		21.2	19.4–23.0	7.0	42.0

Table 1* presents tumor volume (cm*^*3*^*) and tumor measurements (mm) in three planes. Automatic Sectra Volume Tool (Sectra Workstation, IDS7, v. 23.1) was used to calculate the volumes.*

**Table 2:** Anatomical classification of the tumors

SIPAP(Knosp) grade	0	1	2	3	4	*Inconclusive*
Suprasellar	6	7	8	35	13	0
Infrasellar	40	26	1			2
Parasellar dx	14	26	15	7	5	2
Parasellar sin	11	23	18	13	2	2
Anterior	62	5				2
Posterior	58	8				3

Table 2* shows how the included tumors were classified anatomically. The SIPAP and Knosp-classifications have been used.*

**Table 3:** Biochemical classification of the tumors

Biochemical diagnosis	N
Acromegaly	7
Gonadotrophin-producing	0
Prolactinoma	2
ACTH-producing	0
TSH-producing	0
NFPA	60
Total	69

Table 3* shows the distribution of biochemical diagnoses of the included tumors. The majority were nonfunctioning pituitary adenoma (NFPA).*

**Table 4:** Distribution of patient – and tumor related risk factors

	N	Mean age (yrs)	Tumor volume (cm^2)	ISP (mmHg)	ISP > 20 mmHg (N)	Invasive (Knosp 3–4) (N)
Total:	69	59 ± 15	5.6 ± 4.9	22.6 ± 8.3	45 (65%)	22 (33%)
Male:	37	61 ± 15	5.4 ± 5.1	24.8 ± 8.7	28 (76%)	14 (39%)
Female:	32	58 ± 15	5.9 ± 4.9	20.0 ± 7.1	17 (53%)	8 (26%)
		*0.51 (t-test)*	*0.73 (t-test)*	*0.013 (t-test)*	*0.049 (ChiSq)*)	*0.25 (ChiSq)*
		Acute apoplexy (N)	Active smokers (N)	Increased bleeding risk (N)	Increased infection risk (N)	
Total:	69	5 (7%)	6 (10%)	16 (23%)	8 (12%)	
Male:	37	1 (3%)	4 (13%)	13 (35%)	3 (8%)	
Female:	32	4 (13%)	2 (7%)	3 (9%)	5 (16%)	
		*0.11 (ChiSq)*	*0.46 (ChiSq)*	*0.012 (ChiSq)*	*0.33 (ChiSq)*	

Table 4* presents the distribution of the analyzed risk factors in the study population. T-tests and chi-square tests were used to identify any differences between male and female patients. Values are means* ± *standard deviations or n (%).*

Mean ISP was 22.6 ± 8.3 mmHg. Male patients had higher mean ISP than female patients (*p* = 0.013, Table 4). Males had more often increased bleeding risk compared with females (*p* = 0.012, t-test). Mean age was higher among those who had increased bleeding risk (*p* = 0.0379, t-test). Higher age was correlated with larger tumor volume (R^2^ = 0.12, *p* = 0.0035, Pearson). Male patients significantly more often had tumors that were difficult to extract due to their consistency (*p* = 0.030).

### Intraoperative events and postoperative complication frequency

In the study population, the incidence of intraoperative events was 67% (*N* = 46) and that of postoperative complications was 49% (*N* = 34). Five patients (7%) had a clinically significant postoperative complication. Complications frequency is presented in Table 5. A secondary finding was that the incidence of tumors that were perioperatively considered not radically resected (23%, *N* = 12) was higher in the group with parasellar invasive tumors (Knosp ≥ 3) compared with in the group with non-invasive tumors (77%, *N* = 41) (*p* = 0.0024, ChiSq).

**Table 5:** Frequency of intraoperative events and postoperative complications

Intraoperative events	N	%
Bleeding	25	36.2%
Exessive bleeding	6	8.7%
Blood transfusion	0	0%
CSF leakage	27	39.1%
Difficult extraction	22	31.9%
Some intraoperative event	46	66.6%
Postoperative complications		
Epistaxis	23	33.3%
Intracranial hematoma	1	1.4%
CSF leakage	5	7.2%
Lumbar drain	2	2.9%
Fistula repair operation	0	0%
Infection	3	4.3%
Neurological deficit	2	2.9%
Headache	3	4.3%
Some postoperative complication	34	49.2%
Clinically significant complication	5	7.2%

Table 5* shows the distribution of intraoperative events and postoperative complications after endoscopic transsphenoidal pituitary surgery. “Some intraoperative events” is defined as one or more of the listed intraoperative events, and “some postoperative complication” is defined as one or more of the listed postoperative complications. “Clinically significant complication” is defined as at least one of the following: intracranial hematoma, CSF leakage requiring lumbar drainage or fistula repair operation, meningitis, and neurological deficit.*

Two patients had a new neurological deficit postoperatively. One patient had a new CN (cranial nerve) III palsy, and one patient developed hemiparesis. Both had very large tumors: 30 and 20 cm^2^, respectively. The patient who developed postoperative hemiparesis had an extensive macroadenoma that was reported hard in its consistency. Postoperatively the patient woke up with moderate right arm hemiparesis which partly improved during the hospital stay. CT and MRI scans were performed showing no hematoma but minor ischemic lesions in the central parts of the frontal lobe, in the area around the residual tumor. Despite no large focal ischemic lesion was found, the conclusion was that symptoms developed due to micro ischemic damage during arduous resection.

One patient in the population had a postoperative hematoma. Of the 3 (4%) patients who had a postoperative infection, all had bacterial sinusitis. No patients in this population had postoperative meningitis.

### Risk factors

The outcomes of intraoperative events and postoperative complications in relation to separate defined risk factors are presented as odds ratios (OR) in Table 6*.* Large tumor volume was associated with an increased risk of having an intraoperative event (OR 1.37, *p* = 0.022). The risk of having extraction difficulties increased with tumor volume (OR 1.28, *p* = 0.026). Male sex was also a risk factor for having a tumor that was considered difficult to extract (OR 10.0, *p* = 0.0042).

**Table 6:** Risk factors for intraoperative events and postoperative complications

Intraoperative events	Some intraoperative event		Bleeding		CSF leakage		Difficulties to extract			
	OR	p	OR	p	OR	p	OR	p		
Risk factors										
Age	0.96	0.15	0.95	0.057	1.01	0.56	0.96	0.0734		
Sex (male)	1.85	0.39	1.85	0.39	0.52	0.39	**10.0**	**0.0042**		
ISP	0.95	0.21	0.97	0.50	0.99	0.85	0.92	0.065		
Elevated risk of infection	7.69	0.11	2.04	0.55	4.61	0.20				
Elevated risk of bleeding	0.51	0.47	1.13	0.89	0.39	0.35	0.30	0.18		
Smoking	1.01	0.98	1.01	0.73	2.55	0.39	0.73	0.84		
Acute apoplexy	2.27	0.57			3.67	0.34				
Tumor volume	**1.37**	**0.022**	1.16	0.14	1.05	0.63	**1.28**	**0.026**		
Parasellar invasive	0.66	0.61	1.15	0.86	2.66	0.23	0.90	0.91		
Postoperative complications	Some postoperative complication		CSF leakage		Epistaxis		Infection		Headache	
	OR	p	OR	p	OR	p	OR	p	OR	p
Risk factors										
Age	0.96	0.068	**0.90**	**0.019**	1.01	0.95	0.956	0.22	0.91	0.15
Sex (male)	1.61	0.55	9.4	0.13	0.60	0.56	0.42	0.48	0.66	0.84
ISP	1.05	0.27	0.96	0.62	**1.10**	**0.035**	1.033	0.60	1.10	0.46
Elevated risk of infection	1.94	0.62			1.49	0.78				
Elevated risk of bleeding	1.54	0.66			0.75	0.78		0.68		
Smoking	1.80	0.40			0.71	0.77	1.81	0.68		
Acute apoplexy									8.20	0.28
Tumor volume	1.06	0.62	1.05	0.72	1.09	0.67	0.0935	0.68	0.82	0.77
Parasellar invasive	1.30	0.75	8.19	0.14	0.62	0.58			2.55	0.66
Intraoperative bleedning	1.58	0.50	1.59	0.69	0.94	0.93	0.71	0.79	1.69	0.77
Intraoperative CSF leakage	1.34	0.67	0.17	0.21	0.30	0.10	3.29	0.34	0.53	0.75
Difficulties with extraction	0.41	0.28			0.90	0.90	1.17	0.91	0.32	0.65

Table 6* shows unit odds ratios (ORs) for the separate risk factors in relation to the different intraoperative events and postoperative complications. The effect likelihood ratio test was used to calculate ORs*

Higher age was shown to decrease the risk of postoperative CSF leakage (OR 0.93, *p* = 0.019). None of the explored intraoperative events showed any significant association with the risk of postoperative complications.

**Fig. 1:**
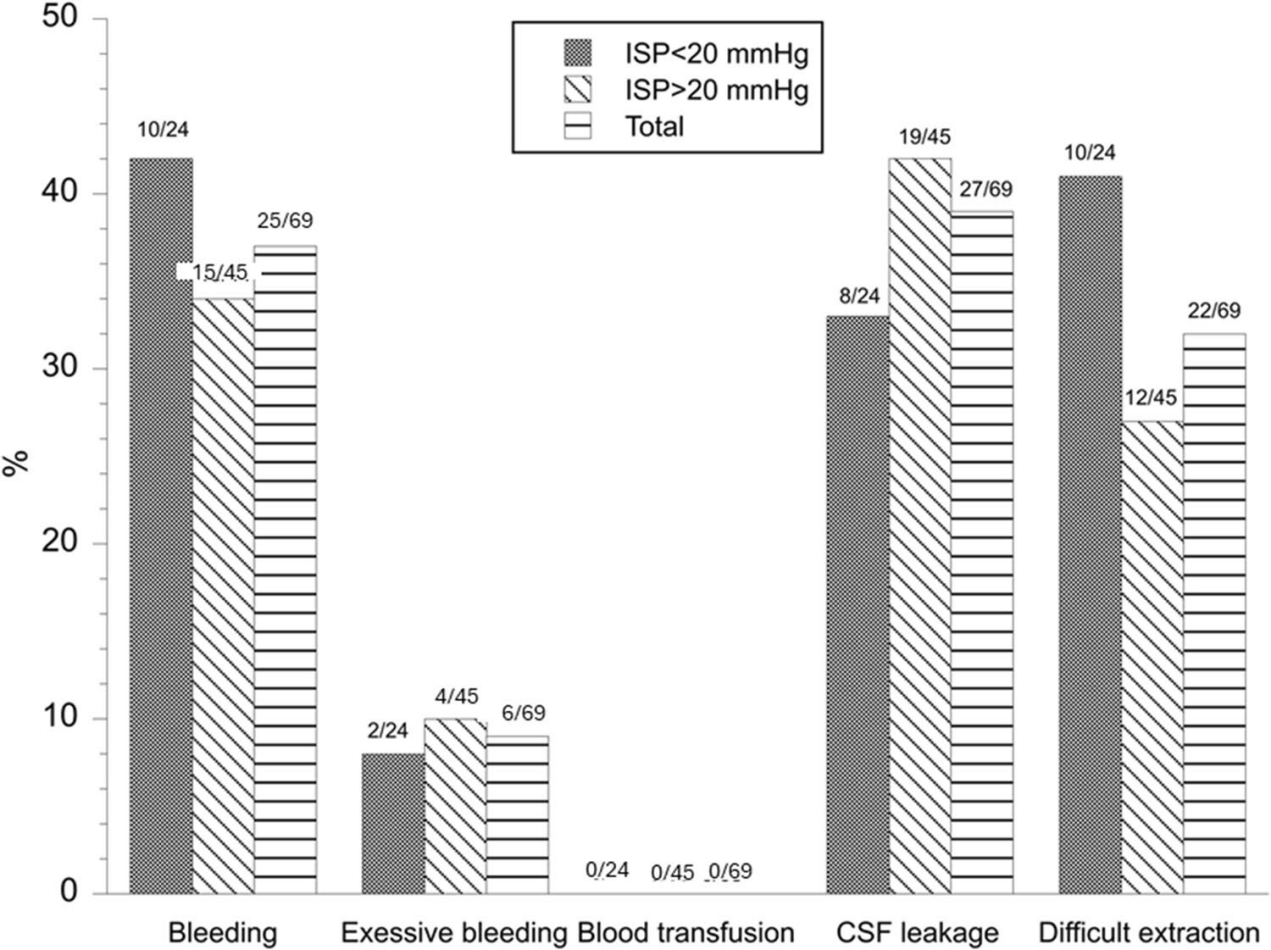
Intraoperative events in patients with high versus low ISP. Figure 1 shows the frequency of intraoperative events in the study population (after exclusions), and in patients with high (≥ 20 mmHg) and low (< 20 mmHg) ISP, respectively. Differences between the groups high and low ISP were analyzed using chi-square tests. No differences in the frequencies of the separate events were found between patients with high and low ISP (*p* > 0.05)

**Fig. 2:**
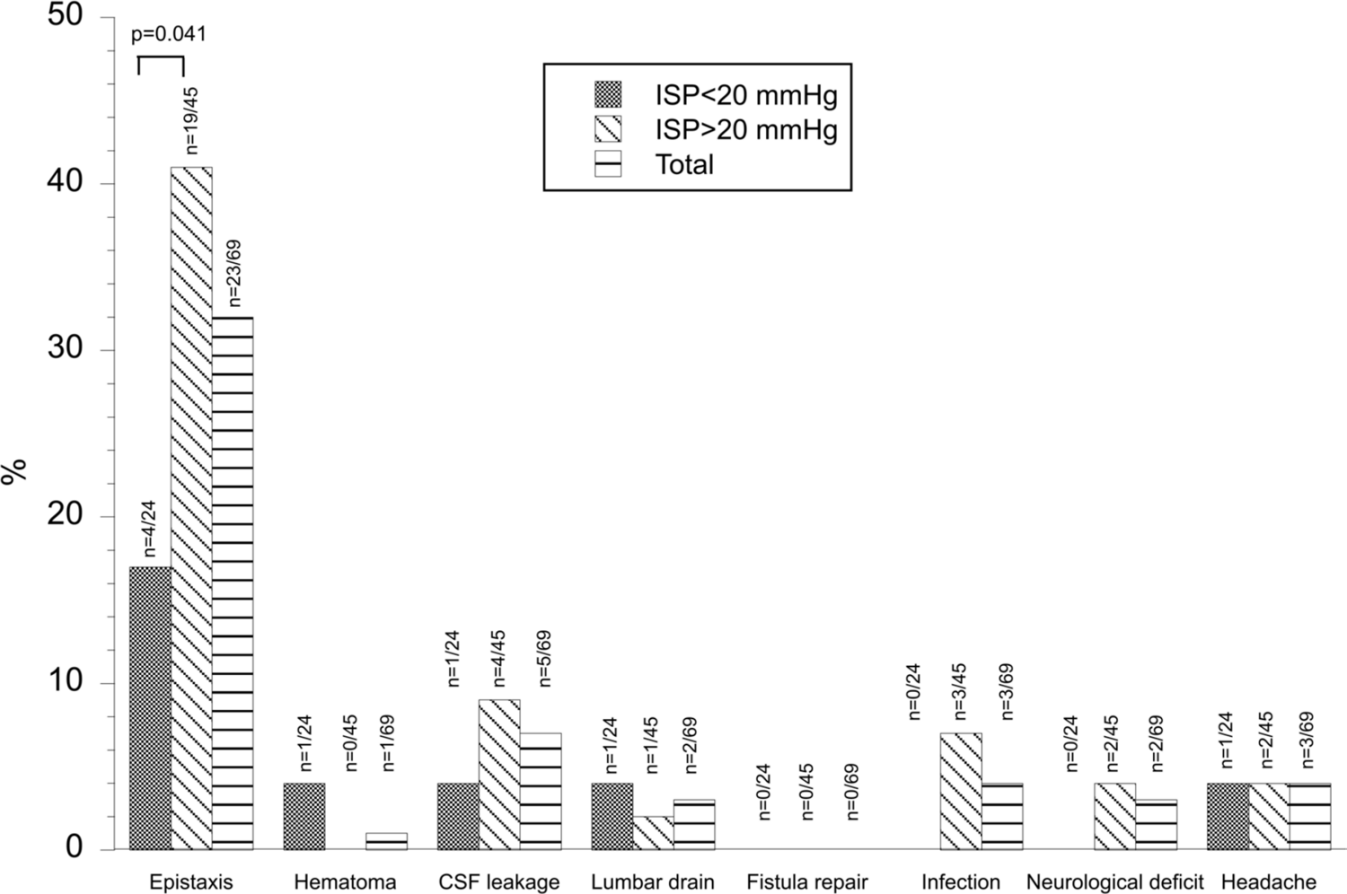
Postoperative complications in patients with high versus low ISP. Figure 2 shows the frequency of postoperative complications in the study population (after exclusions), and in patients with high (≥ 20 mmHg) and low (< 20 mmHg) ISP, respectively. Differences between the groups high and low ISP were analyzed using chi-square tests. The frequency of postoperative epistaxis was higher in the group of patients with high ISP compared with low ISP (*p* = 0.041)

After dichotomizing the population into the groups high ISP (≥ 20 mmHg) versus low ISP (< 20 mmHg), we compared the complication frequency between these groups. Results are presented in Fig. 1 for intraoperative events and Fig. 2 for postoperative complications. Patients with high ISP had an increased frequency of postoperative epistaxis in comparison with patients with low ISP (*p* = 0.041) (Fig. 2).

Male patients with high ISP had increased frequency of bleeding during operation (*p* = 0.011, chi-squared test). No other differences in complication frequency were found between patients with high and low ISP.

## Discussion

We have reported intraoperative events and postoperative complications and explored different risk factors for complications in ETS, with special focus on ISP. Our results suggest tumor volume to be a risk factor for intraoperative events. High age may decrease the risk of postoperative CSF leakage. ISP seems to play a limited role in the incidence of intraoperative events and complications soon after surgery. Two patients who had new neurological symptoms postoperatively had large tumor volumes. We observed no significant association between intraoperative events and postoperative complications.

### Tumor volume

An earlier meta-analysis has suggested large tumor volume as a risk factor for complications in general. For intraoperative CSF leakage, intraventricular extension has also been defined as a risk factor [18]. This is a parameter we have not included in our analysis. One could hypothesize that intraventricular extension somehow reflects tumor volume. When it comes to intraoperative bleeding and tumor anatomy, one study has indicated that growth into the anterior cranial fossa, which may reflect the anterioposterior diameter, is a risk factor for intraoperative intracranial complications including intracranial bleeding [5]. The interpretation of intraoperative bleeding is problematic when comparing the results. It should be noted that our definition of bleeding, which we link to tumor diameter, is based on surgeon reports of intraoperative bleeding, and should not be interpreted as radiologically defined intracranial bleeding.

### Age

Earlier studies have shown high age to be a risk factor for surgical complications in general [18]. However, the finding of low age as a risk factor for postoperative CSF leakage has been presented in a few previous publications [5, 7, 11]. A feasible explanation could be that surgeons are more prone to be aggressive in achieving gross total resection in younger patients than in older, thus increasing the risk of CSF leakage. However, it should be mentioned that this was shown only in postoperative CSF leakage. In our present study, we did not find any correlation between intraoperative and postoperative CSF leakage.

### CSF leakage

We used a broad definition of intraoperative events and postoperative complications. Many of these, such as CSF leakage and some intraoperative bleeding, are expected in ETS and thus have not been defined as adverse events or complications in previous publications. A systematic review found that postoperative CSF leakage and serious bleeding were among the most common postoperative complications [18]. As regards postoperative CSF leakage, our 7.2% overall incidence and 2.9% need for lumbar drainage appear low in comparison to the incidence rates in the review, varying between 1.4% and 16.9%. At our clinic, sealing of the sellar wall (without a primary nasoseptal flap) was performed as a routine, even if no visible CSF leakage was noted during the procedure. A nasoseptal flap was used only in secondary surgery when CSF leakage had occurred postoperatively.

### ISP

The difference in ISP between male and female patients has been presented and discussed in earlier publications based on the same study population [24, 25]. No theoretical explanation to this result has been suggested, nor have similar results been shown in the few earlier studies in this field. When complications were analyzed among male and female patients separately, some weak significances could be seen. These findings should, in our opinion, be considered as mainly the result of small subgroups and therefore be interpreted very cautiously. In one previous study, female sex has been showed to be a risk factor for postoperative CSF leakage [5], a result that was not seen in this study.

We have previously shown that a pressure rise in the sella turcica can be linked to tumor volume and invasiveness [24]. ISP also seems to have the potential to affect the pituitary circulation and thus give rise to endocrinological disorders [25]. This fact gives cause to test the hypothesis that ISP elevation could negatively influence the microenvironment and thus increase the risk of intraoperative events and postoperative complications. The hypothetical background of a potential link between ISP and increased risk of complications would be that the microcirculation is impaired in the pituitary gland and surrounding structures. The pressure levels demonstrated in this study could affect capillary function and venous blood flow, and thus have negative effects on cell function and increase the risk of bleeding. This theory is strengthened by the fact that we have previously demonstrated that elevated ISP has the potential to affect pituitary hormonal function [25]. We chose 20 mmHg tissue pressure as the cut-off value, as this is a well-established threshold, above which many organ systems (including the brain[21]) may be dangerously affected. This is probably due to the capillary and venous circulation becoming seriously affected, resulting in microcirculatory distress. In cases of hormonal disturbances seen in patients with high ISP, we have concluded that the prognosis of recovery after decompressive surgery is good. Our results further suggest a satisfactory postoperative recovery of the microcirculation in the sellar area.

### Epistaxis

There was an increased frequency of epistaxis in patients with high ISP. And in the multivariate regression analysis, ISP appears to increase the risk of epistaxis. Many factors not considered in this study might also affect the risk of postoperative epistaxis. These include, for example, nasal manipulation and compliance with postoperative restrictions. Hypertension has previously been shown to increase the risk of epistaxis [22]. However, hypertension is probably not a potential cause of epistaxis in the present study, as continuous blood pressure monitoring was mandatory for all patients during at least the first 12 h after surgery. The postoperative routine at the clinic was to aggressively treat hypertension, with the goal of systolic blood pressure below 160 mmHg.

Lack of definition of intraoperative events and postoperative complications, including epistaxis, is an issue in previous studies. In a systematic review of postoperative complications based on six studies, the incidence of epistaxis differed from that in our findings [18]. The mean incidence rate for epistaxis was 3.28 ± 3.70% – in stark contrast to our 32%. We believe that this difference is due to reporting bias, as we reported all cases of epistaxis, including those after nasal examination and manipulation postoperatively.

### Bleedings

Our 1.5% incidence of intracerebral hematoma is at the lower end of the presented 0–4.8% incidence of serious bleeding [18].

### Infections

Rhinosinusitis has been reported as a very common postoperative complication, with an incidence as high as 11% [3]. However, the definition of rhinosinusitis is somewhat problematic, as the diagnosis is based on nasal discomfort or observation of nasal crusting, and not strictly limited to confirmed bacterial infection. In this study, we chose a stricter definition of postoperative infection in the head-neck area and showed a 4% risk of postoperative infection – all were sinusitis cases. The incidence of rhinosinusitis after ETS is an important aspect, as it is quite seldom addressed, despite potentially having a high impact on quality of life. In our analyzed population of 69 patients, we did not see any postoperative meningitis, which could seem peculiar. However, the reported incidence varies between 0 and 9%, and as in other complication analyses, definitions often differ between reports [18].

### Strengths and limitations

All studies have merits and weaknesses. This study has one of the largest study populations with ISP data and is the first to present results on ISP in relation to surgical events and complication rates. The material was collected consecutively in a single center covering a large geographical area, which are all factors limiting selection bias. The operations, including ISP measurements, were done in accordance with standard procedures, and in line with what has been described in earlier studies. This maximizes uniformity. Although it is impossible to exclude some pressure drop from the tiny dural opening in conjunction with measurement, results can be compared as long as the technique is uniform. All patients followed a similar clinical routine for postoperative care. One of the limitations of our study is that it contains retrospective data, which may increase the risk of imprecise data interpretation from medical records. However, the surgeon responsible was the same for all patients and intraoperative events and problems were documented immediately in the electronic patient files, as a routine. A new evaluation was performed within hours after finishing the surgery and documented by the surgeon. The day after surgery, another new evaluation was performed and documented. Given this approach, we believe that the preoperative and early postoperative information on events and complications is sound. Our neurosurgical center is the only one in northern Sweden and all postoperative complications were discussed with us. This ensures that information about any complications is found in our electronic patient files and easily retrieved. This further strengthens the validity of our data.

Previous studies of potential risk factors for postoperative complications have not been able to give satisfactory answers regarding such risk factors. Therefore, every contribution to this field may be important. Only a few earlier studies have explored ISP at all, and none have assessed whether pressure in the sella turcica may impact surgical outcome.

## Conclusion

Our study confirms earlier findings of low age as a possible risk factor for postoperative CSF leakage after endoscopic surgery for pituitary adenoma. For intraoperative events, tumor volume seems to be a risk factor. The study does not show any correlation between intraoperative events and postoperative complications. ISP seems to have a limited effect on the risk of intraoperative events or postoperative complications in ETS. Predictive risk factors for surgical complications are still not satisfactorily explained and difficulties with definitions of complications remain an issue in this context.

## Data Availability

Original data are held by the authors and are available on request.

## Abbreviations

CSF: Cerebrospinal fluid
ISP: Intrasellar pressure
ICP: Intracranial pressure
ETS: Endoscopic transsphenoidal surgery
CN: Cranial nerve
NFPA: Nonfunctioning pituitary adenoma
